# Supplementary material for: Inflammation and Venous Thromboembolism in Hospitalized Patients with COVID-19
Source: Diagnostics (Basel). 2023 Nov 19;13(22):3477. doi: 10.3390/diagnostics13223477 (PMC10670045; doi:10.3390/diagnostics13223477)
Supplement: Supplementary file 1 [file diagnostics-13-03477-s001.zip › diagnostics-2572158-supplementary.pdf]

Category	Variables	Description	Values	SI units
**Personal Information**				
	Date of admission	Determines patient's date of admission	day/month/year	NA
	Date of Discharge	Determines patient's date of discharge	day/month/year	NA
	Patient Study ID	Unique numeric code for each patient	Numerical	NA
	Gender	Gender of the patient	Male<br>Female	-
	Age	Age of the patient at admission	Numerical<br>> 16	years
	BMI	Body mass index of the patient	Numerical	kg/m^2
**Past medical history/ Comorbidities**				
	CAD	Past cardiac medical history, type: coronary artery disease, myocardial Infraction, prior cardiac catheterization, self-reported or by patient's medication or else documented (visits at clinic, other documents)	no<br>yes	-
	Hyperlipidemia	Past medical history of the patient, self-reported or by patient's medication or else documented (visits at clinic, other documents)	no<br>yes	-
	Arterial Hypertension	Past medical history of the patient, self-reported or by patient's medication or else documented (visits at clinic, other documents)	no<br>yes	-
	Diabetes mellitus	Past medical history of the patient, self-reported or by patient's medication or else documented (visits at clinic, other documents)	no<br>yes	-
	Cancer	Past medical history of the patient of known cancer, self-reported or by medication patient was receiving, or else documented (visits at clinic, other documents)	no<br>yes	-
	Autoimmune disease	Past medical history of the patient of autoimmune disease, self-reported of by medication patient was receiving (visits at clinic, other documents)	no<br>yes	-
	Pulmonary disease	Past medical history of the patient of chronic pulmonary	no<br>yes	-

		disease		
	CKD	Chronic kidney disease, a type of renal medical history, self-reported or by patient's medication or else documented (visits at clinic, other documents)	no<br>yes	-
	Thyroid disease	Thryoid disease, a type of endocrine medical history self-reported or by patient's medication or else documented (visits at clinic, other documents)	no<br>yes	-
	Morbid Obesity (categorical)	Defined by BMI values	< 35<br>> 35<br>no<br>yes	Kg/m^2
	Dementia	Medical history of dementia	no<br>yes	-
	Smoking	Current smoker	no<br>yes	-
**Hospital Summary**				
	CTBoD	Burden of disease in Computed Tomography as affected lung parenchyma	Numerical	%
	CTBoD (categorical)	Burden of disease in Computed Tomography as affected lung parenchyma	> 50<br>< 50	%
	Duration of symptoms	Days of reported symptoms prior to hospitalization	Numerical	days
	Days of hospitalization	Hospital LoS: days to discharge or death	Numerical	days
	Days to death	Days to death	Numerical	days
	FiO2	Fraction of inspired O2	Numerical	%
	pO2	Partial pressure of O2	Numerical	mmHg
	PFR	PO_2/FiO_2 ratio on admission	Numerical	-
	PFR-150	PO_2/FiO_2 ratio on admission	< 150<br>> 150	-
	PFR-300	PO_2/FiO_2 ratio on admission	< 300<br>> 300	-
**Laboratory parameters**				
	aPTT	Activated Partial Thromboplastin Time	Numerical	sec
	CRP	C-reactive protein serum concentration	Numerical	mg/L
	CRP (categorical)	C-reactive protein serum concentration	< 100<br>> 100	mg/L
	CRP/HDL-C	CRP/HDL-C ratio	Numerical	-
	D-dimers	D-dimer plasma concentration	Numerical	μg/mL
	D-dimers (categorical)	D-dimer plasma concentration	< 2<br>> 2	μg/mL

	Ferritin	Ferritin serum concentration	Numerical	ng/mL
	Ferritin (categorical)	Ferritin serum concentration	< 335<br>> 335	ng/mL
	Fibrinogen	fibrinogen	Numerical	mg/dL
	Fibrinogen (categorical)	fibrinogen	> 600<br>< 600	mg/dL
	HDL-C	serum HDL-Cholesterol	Numerical	mg/dL
	IL-6	Interleukin 6 serum concentration	Numerical	pg/mL
	IL-6 (categorical)	Interleukin 6 serum concentration	< 24<br>> 24	pg/mL
	LDH	Lactate dehydrogenase serum concentration	Numerical	IU/L
	LDH (Categorical)	Lactate dehydrogenase serum concentration	< 230<br>> 230	IU/L
	LDL-C	serum LDL-Cholesterol	Numerical	mg/dL
	Leukocytosis	Leukocytes count	> 11,000	#/μL
	Lymphopenia (categorical)	Lymphocytes count	< 1,000	#/μL
	Lymph/HDL-C	Lymph/HDL-C ratio	Numerical	-
	Neut/HDL-C	Neut/HDL-C ratio	Numerical	-
	Neut/Lymph ratio	Neutrophils to Lymphocytes ratio of the patient	Numerical	-
	Neutr/Lymph ratio (Categorical)	Neutrophils to Lymphocytes ratio of the patient, categorical	< 3.1<br>> 3.1	-
	Neutrophis	White blood cell count of the patient, absolute count	Numerical	#/μL
	Platelet Count	Platelet count of the patient	Numerical	#/μL
	Procalcitonin	Procalcitonin serum concentration	Numerical	ng/mL
	Procalcitonin (categorical)	Procalcitonin serum concentration	< 0.5<br>> 0.5	ng/mL
	T-C	serum Total Cholesterol	Numerical	mg/dL
	Thrombocytopenia (categorical)	Platelets count	< 150000	#/μL
	TRG	serum Triglycerides	Numerical	mg/dL
	TRG/HDL-C	TRG/HDL-C ratio	Numerical	-
	TRG/HDL-C (categorical)	TRG/HDL-C ratio	< 2.5<br>> 2.5	-
	TyG	TyG index	Numerical	-
	TyG 1 (categorical)	TyG index	< 8.7<br>>8.7 and < 9.1	-
	TyG 2 (categorical)	TyG index	< 9.1<br>> 9.1	-
	TyG 3 (categorical)	TyG index	< 8.7<br>> 9.1	-
**Outcomes**				
	VTE	Venous thromboembolism was considered the radiographic evidence of pulmonary	no<br>yes	-

		embolism and vascular tree-in-bud		
	LoS (categorical)	Hospital length of stay: days to discharge or death	< 7<br>> 7	days
	Intubation/Mechanical ventilation (categorical)	Need for invasive mechanical evntilation during hospitalization	no<br>yes	-
	Death (categorical)	Patient death during hospitalization, general COVID-19 ward and/or ICU	no<br>yes	-

Table S1. Definitions and Dictionary of variables in study's registry database.
